# Influences of Microwave-Assisted Extraction Parameters on Antioxidant Activity of the Extract from *Akebia trifoliata* Peels

**DOI:** 10.3390/foods10061432

**Published:** 2021-06-21

**Authors:** Min Luo, Dan-Dan Zhou, Ao Shang, Ren-You Gan, Hua-Bin Li

**Affiliations:** 1 Guangdong Provincial Key Laboratory of Food, Nutrition and Health, Department of Nutrition, School of Public Health, Sun Yat-Sen University, Guangzhou 510080, China; luom65@mail2.sysu.edu.cn (M.L.); zhoudd6@mail2.sysu.edu.cn (D.-D.Z.); shangao@mail2.sysu.edu.cn (A.S.); 2 Research Center for Plants and Human Health, Institute of Urban Agriculture, Chinese Academy of Agricultural Sciences, Chengdu 610213, China; ganrenyou@caas.cn; 3 Key Laboratory of Coarse Cereal Processing (Ministry of Agriculture and Rural Affairs), Sichuan Engineering and Technology Research Center of Coarse Cereal Industrialization, Chengdu University, Chengdu 610106, China

**Keywords:** microwave-assisted extraction, *Akebia trifoliate*, peel, response surface methodology, antioxidant capacity, total phenolic content, HPLC

## Abstract

*Akebia trifoliata* is a fruit with rich nutritional properties, and its peel is produced as a by-product. In this research, we investigated the influences of microwave-assisted extraction parameters on antioxidant activity of the extract from *Akebia trifoliata* peels, and the ferric-reducing antioxidant power (FRAP) and Trolox equivalent antioxidant capacity (TEAC) as well as total phenolic contents (TPC) were used to optimize extraction parameters. The influences of ethanol concentration, microwave power and solvent-to-material ratio, as well as extraction temperature and time on TPC, FRAP and TEAC values, were assessed using single-factor tests. Three parameters with obvious effects on antioxidant capacity were selected to further investigate their interactions by response surface methodology. The optimal extraction parameters of natural antioxidants from *Akebia trifoliata* peels were ethanol concentration, 49.61% (*v*/*v*); solvent-to-material ratio, 32.59:1 mL/g; extraction time, 39.31 min; microwave power, 500 W; and extraction temperature, 50 °C. Under optimal conditions, the FRAP, TEAC and TPC values of *Akebia trifoliata* peel extracts were 351.86 ± 9.47 µM Fe(II)/g dry weight (DW), 191.12 ± 3.53 µM Trolox/g DW and 32.67 ± 0.90 mg gallic acid equivalent (GAE)/g DW, respectively. Furthermore, the main bioactive compounds (chlorogenic acid, rutin and ellagic acid) in the extract were determined by high-performance liquid chromatography. The results are useful for the full utilization of the by-product from *Akebia trifoliate* fruit.

## 1. Introduction

**Figure 1:**
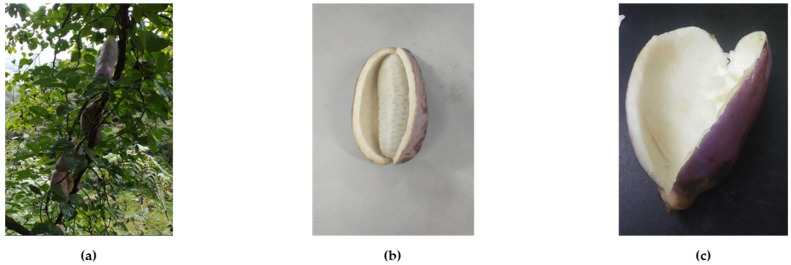
The vine (**a**), fruit (**b**) and peel (**c**) of *Akebia trifoliate*.

*Akebia trifoliate* is a fruit and belongs to the family Lardizabalaceae. It looks like a bunch of thick bananas (Figure 1b), and ripe fruit could split longitudinally. In recent years, *Akebia trifoliate* has been widely cultivated because of its commercialization [1]. It contains a variety of bioactive ingredients, exerting diuretic, antibacterial, antioxidant, anti-inflammatory and anticancer activities [2,3,4]. In the Chinese Pharmacopoeia, the stems and fruits of *Akebia trifoliate* are recorded as herbal medicines [2]. In addition, it was demonstrated that *Akebia trifoliate* seed extract could inhibit cell proliferation and have anticancer potency in hepatocellular carcinoma cells [5]. The pulp of *Akebia trifoliate* is edible, delicious and nutritious, and could also be processed into commodities such as desserts, juices and vinegar, but its peel is not edible and accounts for a large proportion of the whole fruit (Figure 1c). With the high consumption of *Akebia trifoliate*, a lot of by-products will be produced and become an environmental problem. The peel contains rich phytochemicals, and is a potential natural source of antioxidants. Therefore, it would be of great value to develop an extraction method for antioxidants from *Akebia trifoliate* peels.

The extraction of antioxidants from plant materials could be carried out using different methods, including traditional techniques such as reflux, percolation, soaking, maceration and Soxhlet extraction, and new extraction methods such as enzyme-assisted extraction, ultrasound-assisted extraction and microwave-assisted extraction (MAE) [6,7,8,9,10]. Taking into account the consumption of organic solvent, extraction time, cost and efficiency, MAE is an advanced method with many advantages [10]. The electromagnetic field generated by microwave radiation could accelerate the movement of molecules, resulting in a rapid extraction [11]. MAE has been used to extract active ingredients from some plants due to its high efficiency, stability and reproducibility [12,13]. In addition, there is no study on the MAE of antioxidants from *Akebia trifoliate* peels in the published literature.

In the present research, the antioxidant activities and total phenolic contents (TPC) of the extract were applied to test the impacts of ethanol concentration, microwave power, solvent-to-material ratio, as well as extraction temperature and time on extraction efficiency of natural antioxidants from *Akebia trifoliate* peels by single-factor tests. Then, three obvious influencing factors were selected to study the interactions of different parameters by response-surface methodology (RSM) with a central composite design. Moreover, major phenolic compounds in the extracts were determined using high-performance liquid chromatography with a photodiode array detector (HPLC-PDAD). The results of this paper may be useful for the value-added exploitation of *Akebia trifoliate* peels.

## 2. Materials and Methods

### 2.1. Sample and Chemicals

The fresh *Akebia trifoliate* fruit was purchased from Haikou, Hainan Province, China, and was washed by water. The peels were obtained by the removal of *Akebia trifoliate* pulp and dried at 35 °C. The dried sample contained 0.67 ± 0.63% moisture and was ground into particles using a conventional grinder. The powder sample was filtered through a 100-mesh sieve and sealed and stored at 4 °C.

Gallic acid, Folin–Ciocalteu’s phenol reagent, 2,2′-azinobis (3-ethylbenothiazoline-6-sulfonic acid) diammonium salt (ABTS), 2,4,6-tri(2-pyridyl)-S-triazine (TPTZ) and 6-hydroxy-2,5,7,8-tetramethylchromane-2-carboxylic acid (Trolox) were obtained from Sigma-Aldrich (St. Louis, MO, USA). The methanol and formic acid were acquired from Macklin Chemical Factory (Shanghai, China), and they were of chromatographic grade. The ethanol, acetic acid, hydrochloric acid, potassium persulfate, iron(II) sulfate heptahydrate, iron(III) chloride hexahydrate, sodium acetate as well as sodium carbonate were acquired from Tianjin Chemical Factory (Tianjin, China) with analytical grade. The standards gallic acid, chlorogenic acid, ferulic acid, rutin, ellagic acid and quercetin were bought from Guangzhou Meilun Biological Co. Ltd. (Guangzhou, China). Deionized water was used throughout the experiment.

### 2.2. Microwave-Assisted Extraction (MAE)

The MAE was carried out using an X-100A microwave equipment (Xianghu Instrumental Co., Beijing, China), and the operating parameters (power, temperature and time) could be adjusted and controlled. The microwave equipment controls temperature by controlling microwave time, not microwave power. When microwave power is fixed, different temperatures can be obtained by changing microwave time, which is automatically adjusted by the microwave equipment. On the other hand, the same temperature can be obtained using different microwave powers by changing microwave time, which is also automatically adjusted by the microwave equipment. Thus, it should be that the microwave time is “apparent” microwave time, not “actual” microwave time. That is, when the expected temperature arrives, the microwave equipment stops working; when the temperature is lower than the expected temperature, the microwave equipment works again until the expected temperature. The powder sample of peel (1.000 g) was put in a tube, and different volumes and concentrations of aqueous ethanol solutions were added. After extraction, the tube was cooled with tap water. Finally, the supernatant was collected after centrifuging with 4600× *g* for 6 min at 4 °C for follow-up experiments.

### 2.3. Maceration Extraction (ME)

The powder sample of peel (1.000 g) was mixed with 32.59 mL of 49.61% ethanol aqueous (*v*/*v*) in a tube, which was shaken in a water bath at 25 °C for 24 h, and the supernatant was collected after centrifugation (4600× *g*, 4 °C, 6 min).

### 2.4. Measurement of Total Phenolic Contents (TPC) and Antioxidant Capacity

The antioxidant capacity, including ferric-reducing antioxidant power (FRAP) and Trolox equivalent antioxidant capacity (TEAC), and TPC in *Akebia trifoliate* peel extracts, were measured based on the literature [6,14,15,16].

#### 2.4.1. Determination of TPC Value

The TPC of the extract was determined using the Folin–Ciocalteu method. The 0.5 mL of diluted sample solution was mixed with 2.5 mL of 0.2 mol/L Folin–Ciocalteu reagent. Additionally, 2 mL of saturated sodium carbonate solution was added after 4 min, and then incubated in the dark for 120 min at room temperature. Subsequently, the absorbance of the mixture was evaluated at 760 nm. Finally, the TPC value was expressed as mg of gallic acid equivalent (GAE)/g of dry weight (DW).

#### 2.4.2. Determination of TEAC Value

ABTS^•+^ stock solution was consisted of 2.45 mmol/L K_2_S_2_O_8_ solution and 7 mmol/L ABTS^•+^ solution (1:1, *v*/*v*). It could be used after 16 h of incubation in the dark, and was used up within 48 h. The absorbance of the stock solution was diluted to 0.71 ± 0.05 at 734 nm, and the obtained dilution ratio was used to prepare the reaction solution. The 0.10 mL diluted sample solution was added to 3.8 mL reaction solution, and the mixture reacted for 6 min in the dark at room temperature, and then the absorbance of the sample was determined at 734 nm. Based on the standard curve obtained from the Trolox solution with known concentrations, the TEAC values were shown as µmol Trolox/g DW of peel.

#### 2.4.3. Determination of FRAP Value

The FRAP working solution was composed of 10 mmol/L of TPTZ solution, 20 mmol/L of FeCl_3_ solution and 300 mmol/L of CH_3_COONa buffer (1:1:10, *v*/*v*/*v*), and then put in a 37 °C water bath for subsequent experiments. The 0.1 mL diluted supernatant was mixed with 3 mL FRAP working solution and reacted at room temperature for 4 min. The absorbance of the mixture was evaluated at 593 nm. The ferrous sulfate was used as a standard, and the FRAP value was shown as µmol Fe(II)/g DW of peel.

### 2.5. Experimental Design and Statistical Analysis

#### 2.5.1. Single-Factor Tests

Influences of five independent variables, namely, ethanol concentration (20–70%, *v*/*v*), microwave power (300–800 W), extraction temperature (30–70 °C), solvent-to-material ratio (10:1–60:1 mL/g) and extraction time (10–60 min) on the extraction efficiency were evaluated. When other factors were controlled, the change of a single factor was observed to study its effect on TPC, FRAP and TEAC values.

#### 2.5.2. RSM

Theoretically, RSM can be applied for optimizing all input variables. Actually, three parameters were usually selected to study interactions in the literature [10], which are often enough, and more parameters are not necessary, and even waste time, cost and labor. Thus, in this study, three parameters showing the strongest effects, including ethanol concentration, solvent-to-material ratio and extraction time, were chosen to test their interactions for extraction of antioxidants from *Akebia trifoliate* peel in the RSM design by Design Expert 11 (Stat-Ease Inc., Minneapolis, MN, USA). The three-variable-five-level central composite design (CCD) matrix involving 20 experiments was performed (Table 1). Furthermore, interactions of two of three tested parameters on response values were displayed using a 3D surface map. Finally, results were acquired under the optimal extraction parameters and compared with the predicted values.

### 2.6. Determination of Main Components in the Extract by HPLC-PDAD

The main components in *Akebia trifoliate* peel extract were determined using HPLC-PDAD (Waters 2996, Milford, MA, USA). The Agilent Zorbax Eclipse XDB-C18 column (4.6 × 250 mm, 5 µm, Santa Clara, CA, USA) was used for gradient elution. The mobile phase consisted of solution A (methanol) and solution B (0.1% formic acid solution). The elution procedure was set as follows: 0−10 min, 2−17% A; 10−15 min, 17−19% A; 15−20 min; 19−22% A; 20−40 min, 22−47% A; 40−50 min, 47−50% A; 50−60 min, 50−58% A; 60−70 min, 58−2% A; and 70−75 min, 2% A. The flow rate was 0.8 milliliter/min, and 20 microliters of the extract were injected into the column. The main phenolic components in *Akebia trifoliate* peel extract were determined by comparing with the retention time and spectra, as well as peak area of the standards. The contents of main components were shown as mg/g DW of peel.

### 2.7. Statistical Analysis

The experimental results were shown as mean ± standard deviation. All data analysis was performed by Design Expert 11 software, SPSS 25.0 statistics software (IBM Corp., Armonk, NY, USA) and Excel 2010. The one-way analysis of variance (ANOVA) was applied to analyze the statistical significance among multiple groups, and the difference was regarded as significant at *p* < 0.05.

## 3. Results and Discussion

### 3.1. Results of Single-Factor Tests

#### 3.1.1. Effect of Ethanol Concentration

**Figure 2:**
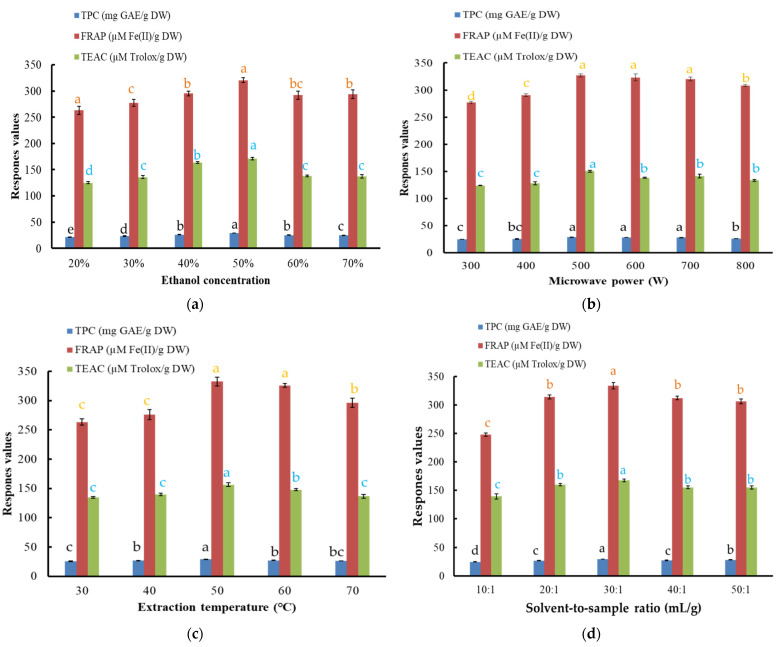

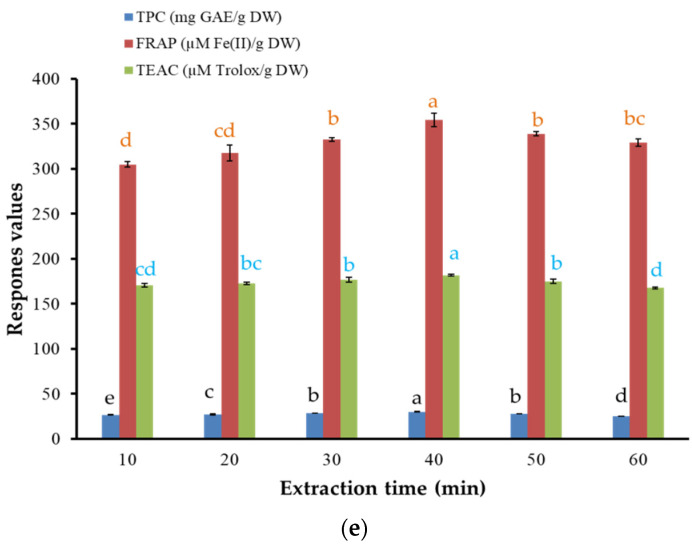
Effects of single factors on the TPC, FRAP and TEAC values of *Akebia trifoliate* peel extract. (**a**), Ethanol concentration; (**b**), microwave power; (**c**), extraction temperature; (**d**), solvent-to-material ratio; (**e**), extraction time. Different colored letters (black, orange and blue) represent significant differences (*p* < 0.05) in TPC, FRAP and TEAC values among different groups, and the same letter illustrates no significant difference (*p* > 0.05).

In this research, aqueous ethanol solution was selected as an extraction solvent because of its easy accessibility and low toxicity, which has been applied to extract natural antioxidants from several plants, such as various fruits, the flower of *Limonium sinuatum* and wild fruit *Melastoma sanguineum* [6,17,18]. First, the effects of different ethanol concentrations on the extraction efficiency of components in *Akebia trifoliate* peel extract were studied under the following conditions: extraction temperature, 50 °C; microwave power, 500 W; solvent-to-material ratio, 30:1 mL/g as well as extraction time, 20 min. The trends of the TPC and antioxidant capacity of the extract under different ethanol concentrations are exhibited in Figure 2a.

FRAP values continued to increase between 20 and 50% (*v*/*v*) of ethanol concentration and then fell with further increase of ethanol concentration (Figure 2a). At the same time, the TPC and TEAC values showed similar trends to the FRAP value, with the maximal values at 50% ethanol (*v*/*v*). The influencing trends of ethanol on TEAC of *Akebia trifoliate* peel extract were also in agreement with those reported in the literature [17]. The TPC, FRAP and TEAC values were 28.90 ± 0.18 mg GAE/g DW, 320.64 ± 4.76 µmol Fe(II)/g DW and 170.98 ± 2.37 µmol Trolox/g DW at 50% ethanol (*v*/*v*), respectively. Therefore, 50% ethanol (*v*/*v*) was chosen for further experiments.

#### 3.1.2. Effect of Microwave Power

Microwave power was one of the vital factors impacting extraction efficiency. The effects of microwave power of 300−800 W on the TPC and antioxidant activity of the extracts were investigated under the parameters of 50% ethanol (*v*/*v*), solvent-to-material ratio of 30:1 mL/g, 50 °C as well as 20 min (Figure 2b).

The FRAP value kept increasing between 300 and 500 W of the microwave power and then fell slowly. The TEAC and TPC values also increased continuously from 300 to 500 W, and then reduced slowly. The changing trends of three parameters of *Akebia trifoliate* peel extract were also in agreement with those reported in a previous paper [10]. MAE is based on heating the sample by utilizing microwave energy. Microwave radiation could destroy cell structure so that the active compound could dissolve into the sample [13]. With the rise of microwave power, the interaction between the sample molecules and the electromagnetic field was enhanced, and the recovery efficiency was improved [10]. However, higher microwave power might result in thermal degradation and deterioration of some active components [19]. Consequently, 500 W was selected as the optimal microwave power.

#### 3.1.3. Effect of Extraction Temperature

The other parameters remained at 50% ethanol (*v*/*v*), 500 W, solvent-to-material ratio of 30:1 mL/g as well as 20 min, the changes of FRAP, TEAC as well as TPC values with extraction temperature are exhibited in Figure 2c.

The influence of extraction temperature on TPC value was weak. Generally, the response values increased as temperature increased from 30 to 50 °C, and decreased slightly when the temperature increased from 50 to 70 °C. The influencing trends of temperature on TEAC of *Akebia trifoliate* peel extract were also similar to those reported in the literature [17]. It was proved that heat produced from the microwave was the significant factor that affected the extraction yields of bioactive compounds [19]. However, excessive temperatures could degrade the active ingredients in the sample. Hence, 50 °C was selected as the optimal extraction condition.

#### 3.1.4. Effect of Solvent-to-Material Ratio

The effects of solvent-to-material ratio on FRAP, TEAC and TPC values were studied when other conditions were fixed at 50% ethanol (*v*/*v*), 500 W, 50 °C and 20 min, and the trends of response values are exhibited in Figure 2d.

The antioxidant ability and TPC value increased when the solvent-to-material ratio increased from 10:1 to 30:1, reached the maximum at 30:1 mL/g and decreased slightly. The changing trends of three parameters of *Akebia trifoliate* peel extract were also similar to those reported in the literature [10]. The solvent-to-material ratio was considered to be one of the most important factors affecting the extraction yields of phenolic compounds [20,21]. A larger ratio of solvent-to-material was conducive to the rapid release of the intracellular substances and enhanced extraction yield [22]. Nevertheless, it was also found that a much large ratio of solvent-to-material would lead to a decline in the recovery efficiency [19]. As a result, 30:1 mL/g was chosen as the optimal solvent-to-material ratio.

#### 3.1.5. Effect of Extraction Time

The effects of extraction time on the recovery efficiency were studied under other conditions of 50% ethanol (*v*/*v*), 500 W, 50 °C and a solvent-to-material ratio of 30:1 mL/g, and results are exhibited in Figure 2e.

FRAP, TEAC and TPC values increased with time until the maximum yield was reached at 40 min, and were 354.39 ± 7.52 µmol Fe(II)/g DW, 181.84 ± 0.73 µmol Trolox/g DW as well as 30.10 ± 0.19 mg GAE/g DW, respectively. However, further prolongation of extraction time resulted in a slow decrease of the response values. This was because more bioactive compounds dissolved into the solvent with the increase of extraction time. However, longer extraction time could produce negative effects due to overexposure to radiation and potential degradation of the bioactive compounds [23]. Thus, 40 min was the optimal extraction time.

### 3.2. Results of RSM Optimization

#### 3.2.1. Results of CCD

Based on single-factor experimental results, ethanol concentration, solvent-to-material ratio, as well, as extraction time were chosen as extraction parameters optimized by RSM because they were the main factors influencing the yields of FRAP and TEAC as well as TPC. The middle levels of central composite design were set as 50% ethanol (*v*/*v*), solvent-to-material ratio of 30:1 mL/g, as well as extraction time of 40 min. In addition, other conditions were set as microwave power of 500 W and extraction temperature of 50 °C. The design of 20 experiments, as well as the actual response values (TPC, FRAP and TEAC), are exhibited in Table 2. For the antioxidant capacity of the extract from *Akebia trifoliate* peels, the FRAP value ranged from 286.31 to 376.76 µmol Fe(II)/g DW as well as TEAC value ranged from 142.66 to 211.60 µmol Trolox/g DW. Besides, the TPC value of *Akebia trifoliate* peel extract ranged from 26.84 to 32.93 mg GAE/g DW.

#### 3.2.2. Model Fitting

Y represents the yields of TPC, FRAP and TEAC of *Akebia trifoliate* peel extract. In addition, X_1_, X_2_, X_3_ are ethanol concentration, solvent-to-material ratio and extraction time, respectively.

Y_FRAP_ =367.2 − 8.28X_1_ + 10.1X_1_X_2_ –16.32X_1_^2^ − 12.78X_2_^2^ − 15.56X_3_^2^ (1)

Y_TEAC_ = 194.18 + 10.06X_2_ + 11.14X_1_X_2_ − 7.7X_1_^2^ − 7.83X_2_^2^ − 9.16X_3_^2^ (2)

Y_TPC_ = 32.18 − 0.83X_1_ + 0.88X_1_X_2_ + 1.04X_2_X_3_ − 0.87X_1_^2^ (3)

The ANOVA in Table 3 showed that three fitted models (TPC, FRAP and TEAC) were all significant (*p* < 0.05), and *F* values were 7.28, 6.52 and 3.88, respectively. In addition, the insignificance of the lack-of-fit items (*p* > 0.05) indicated that the selected models were suitable. The R^2^ values of FRAP, TEAC and TPC fitting models were 0.8676, 0.8544 as well as 0.7773, respectively, implying that most of the variations of response values were attributed to three independent variables. In addition, coefficients of variation (C.V.%) of FRAP, TEAC and TPC were 3.78, 5.42 and 3.32, suggesting that all experimental data had high accuracy and sufficient reliability. These results all indicated the validity of the models.

The effects of ethanol concentration (X_1_), solvent-to-material (X_2_) and extraction time (X_3_), on the FRAP, TEAC and TPC of *Akebia trifoliate* peel extract, are exhibited in Equations (1)–(3) as well as Table 4. For FRAP value, the linear term of ethanol concentration as well as its quadratic term (X_1_ and X_1_^2^), the quadratic terms of solvent-to-material ratio (X_2_^2^) and extraction time (X_3_^2^) had significant negative influences (*p* < 0.05). The interaction terms of ethanol concentration with solvent-to-material ratio (X_1_X_2_) had significant positive influences (*p* < 0.05). For TEAC value, the linear term of solvent-to-material ratio (X_2_), and its interaction term with ethanol concentration (X_1_X_2_) had a very significant positive effect (*p* < 0.01), but the quadratic terms of ethanol concentration (X_1_^2^), solvent-to-material ratio (X_2_^2^) and extraction time (X_3_^2^) had significant negative effects (*p* < 0.05). For TPC value, the ethanol concentration (X_1_), as well as its quadratic term (X_1_^2^), had significant positive effects (*p* < 0.05), while the interaction terms of the solvent-to-material ratio with the ethanol concentration (X_1_X_2_) and extraction time (X_2_X_3_) had significant negative effects (*p* < 0.05).

#### 3.2.3. Analysis of Response Surface Plots

**Figure 3:**
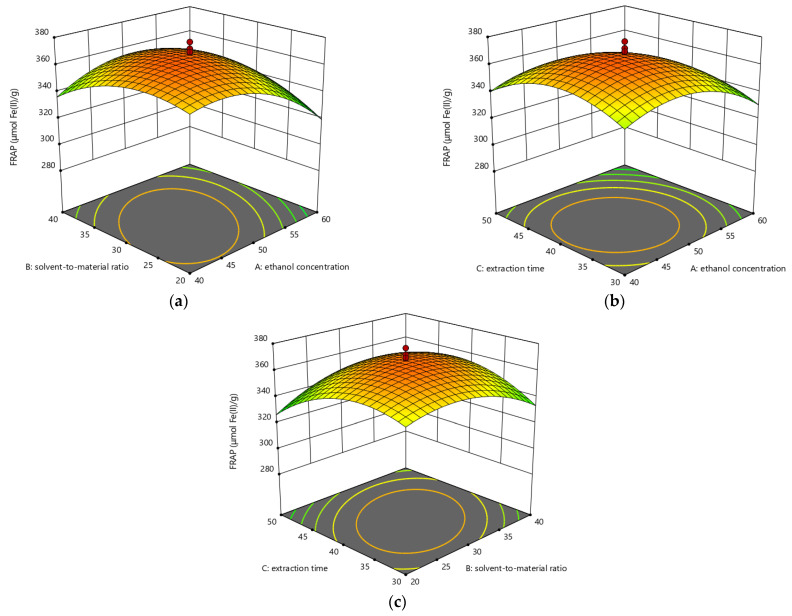
The three-dimensional response surface plots for the influences of ethanol concentration/solvent-to-material ratio (**a**); ethanol concentration/extraction time (**b**); as well as solvent-to-material ratio/extraction time (**c**) on the FRAP values.

**Figure 4:**
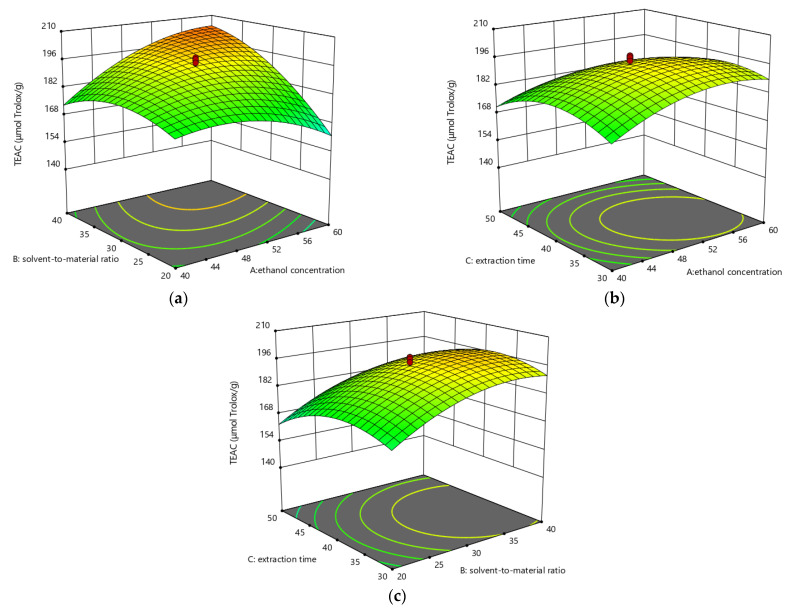
The three-dimensional response surface plots for the effects of ethanol concentration/solvent-to-material ratio (**a**); ethanol concentration/extraction time (**b**); as well as solvent-to-material ratio/extraction time (**c**) on the TEAC values.

**Figure 5:**
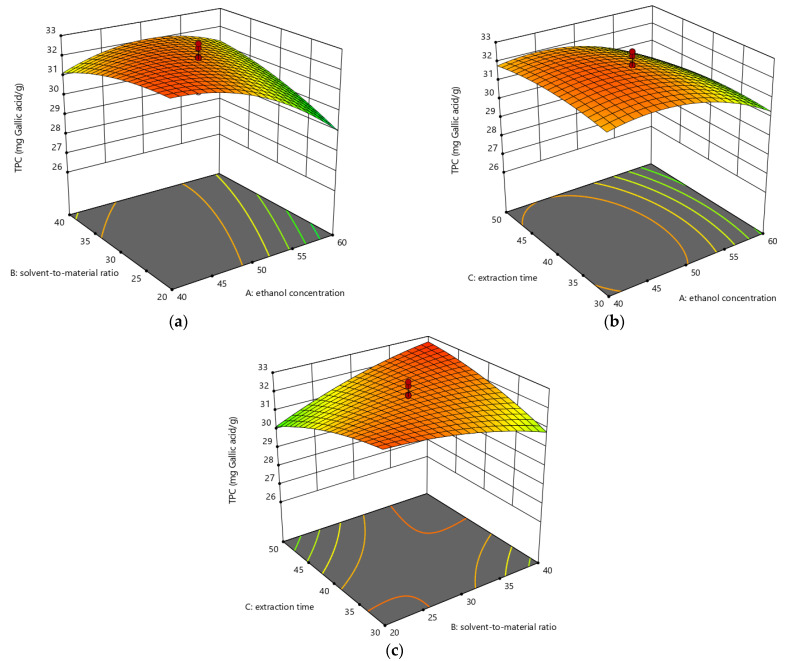
The three-dimensional response surface plots for the effects of ethanol concentration/solvent-to-material ratio (**a**); ethanol concentration/extraction time (**b**); as well as solvent-to-material ratio/extraction time (**c**) on the TPC values.

The relationships between selected parameters and response values are described in Figure 3, Figure 4 and Figure 5.

As seen from Figure 3a, when the extraction time was controlled at 40 min, the FRAP value of *Akebia trifoliate* peel extracts first increased with the rises of ethanol concentration and solvent-to-material ratio, and then decreased when the ethanol concentration and solvent-to-material ratio exceeded approximately 50% (*v*/*v*) and 30:1 mL/g, respectively. In addition, the decreasing trend of the FRAP value with ethanol concentration was more obvious than that of the FRAP value with solvent-to-material ratio. Besides, the elliptical contour plot suggested that there was a strong interaction between ethanol concentration and solvent-to-material ratio. When the solvent-to-material ratio was constant (30:1 mL/g), the effect trend of ethanol concentration on the FRAP value in Figure 3b was similar to that in Figure 3a. For the effect of extraction time, it also showed a parabolic trend on the FRAP value. When ethanol concentration remained 50% (*v*/*v*), the FRAP value showed an increasing trend with the increase of extraction time and solvent-to-material ratio and reached the maximum at about 40 min of extraction time and 30:1 mL/g of solvent-to-sample ratio (Figure 3c).

The relationship between the TEAC value and three independent factors is displayed in Figure 4. Figure 4a exhibited that the TEAC value gradually increased when ethanol concentration rose from 40% to about 50% (*v*/*v*) and solvent-to-material ratio rose from 20:1 to about 30:1 mL/g as extraction time was fixed at 40 min. When the ethanol concentration and solvent-to-material ratio exceeded about 50% (*v*/*v*) and 30:1 mL/g, respectively, the TEAC value gradually decreased. In addition, the interaction between ethanol concentration and the solvent-to-material ratio was strong. In Figure 4b, the TEAC value changed slightly with ethanol concentration or extraction time when the solvent-to-material ratio was controlled at 30:1 mL/g. Combined with ANOVA, the change degree of the TEAC value with extraction time was greater than that of the TEAC value with ethanol concentration. In Figure 4c, under fixed 50% ethanol (*v*/*v*), effects of solvent-to-material ratio and extraction time on the TEAC yield were similar to those exhibited in Figure 4a,b. Since the linear and quadratic terms of solvent-to-material ratio were significant, the TEAC value of the extracts was mainly affected by the solvent-to-material ratio.

The pairwise interactions of three parameters are exhibited in Figure 5. As seen from Figure 5a, when extraction time was controlled at 40 min, the TPC yield of the extracts increased slightly with ethanol concentration increasing from 40% to approximately 45% (*v*/*v*), and then dropped when the ethanol concentration increased further. In addition, the influence of the solvent-to-material ratio on the TPC value was relatively gentle. Besides, the contour plots showed that the interaction between ethanol concentration and solvent-to-material ratio had a significant effect on the TPC value. Figure 5b showed that when the solvent-to-material ratio was controlled at 30:1 mL/g, the effect of extraction time on the TPC value was relatively weak, and influences of ethanol concentration on the TPC value were greater than that of extraction time, indicating that the TPC value was mainly impacted by ethanol concentration. In Figure 5c, combined with ANOVA, there was a significant interaction between extraction time and solvent-to-material ratio on the TPC value.

#### 3.2.4. Verification of Optimal Extraction Conditions

Based on the results from one-way ANOVA analysis as well as Equations (1)–(3), the optimal extraction conditions were as follows: ethanol concentration, 49.61% (*v*/*v*); solvent-to-material ratio, 32.57 mL/g; extraction time, 39.31 min; extraction temperature, 50 °C; and microwave power, 500 W. It should be pointed out that these data were theoretical values, and the nearest values should be used according to the controllable precision of equipment in actual conditions. Under optimal extraction parameters, the TPC, FRAP and TEAC values of *Akebia trifoliata* peel extract were 33.17 ± 0.62 mg GAE/g DW, 356.51 ± 3.06 µM Fe(II)/g DW as well as 191.12 ± 3.53 µM Trolox/g DW, respectively (Table 5), and they were increased by 55.44%, 35.56%, 53.01% for TPC, FRAP and TEAC values, respectively, when compared with the values obtained under the initial conditions. These actual values were close to the predicted values of 32.23 mg GAE/g DW, 196.18 µM Trolox/g DW and 366.59 µM Fe(II)/g DW, which proved the accuracy and reliability of the fitted model. In addition, a conventional maceration extraction method was performed for extracting components from *Akebia trifoliata* peels, and the TPC, FRAP and TEAC values were 26.28 ± 0.38 mg GAE/g DW, 297.66 ± 1.37 µM Fe(II)/g DW and 149.54 ± 1.87 µM Trolox/g DW, respectively. In summary, MAE improved the extraction efficiency and increased the extraction rate by 26.22%, 27.81% and 19.77%, respectively. In other words, the recovery rates of TPC, FRAP and TEAC values were 126.22%, 127.81% and 119.77%, respectively, when their values, obtained using a conventional maceration extraction method, were defined as the initial values.

### 3.3. Identification of Phenolic Compounds in Akebia trifoliate Peel Extract by HPLC Method

The bioactive components in natural products are of great interest to researchers because of multiple health benefits and no or minimal side effects. Antioxidants, especially phenolic compounds, are usually the main contributors to antioxidant capacity and other biological activities of natural products. Some bioactive components that were the main phenolic compounds in *Akebia trifoliate* peels have been isolated and identified in the literature [3,4]. In addition, bioactive components in many fruits and their peels have been determined in our previous papers [6,24], where an analytical method developed by Sakakibara and his colleagues was adopted [25]. Therefore, the extract from *Akebia trifoliate* peels was first screened to find out major/predominant phenolic compounds using the method reported in the literature, where approximately 100 compounds in vegetables, fruits and teas could be screened [25]. The compounds obtained by this method were combined with the compounds reported in the literature [3,4] to select major/predominant phenolic compounds and their standards to quantify the contents of compounds in the *Akebia trifoliate* peel extract.

**Figure 6:**
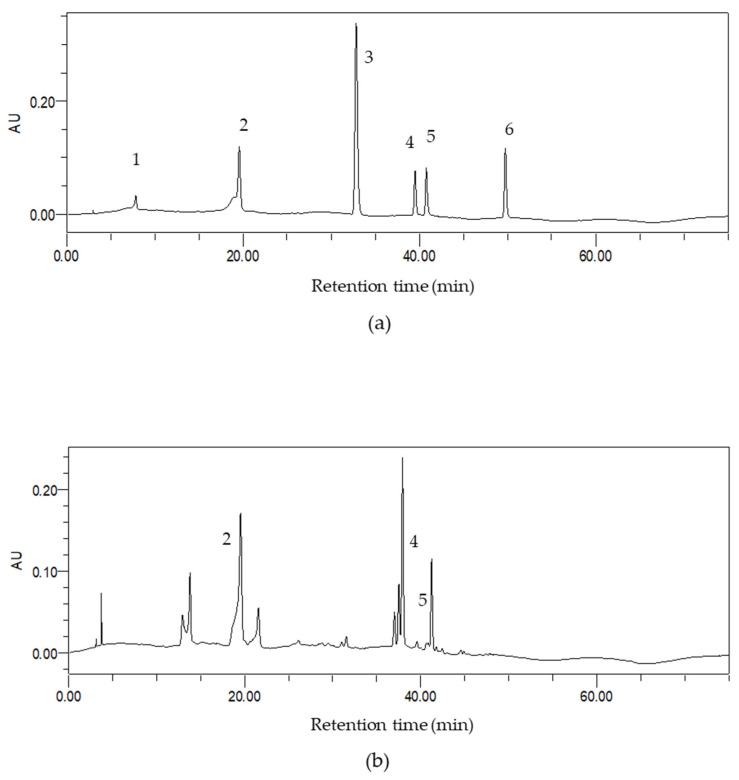
The chromatograms of high-performance liquid chromatography with diode array detection at 310 nm of standards (**a**); as well as *Akebia trifoliate* peel extract (**b**). 1, gallic acid; 2, chlorogenic acid; 3, ferulic acid; 4, rutin; 5, ellagic acid; 6, quercetin.

The bioactive components in the extract were determined by the HPLC method (Figure 6), and the contents of three compounds (chlorogenic acid, rutin and ellagic acid) were 6.38 ± 0.32 mg/g DW, 7.31 ± 0.17 mg/g DW as well as 3.41 ± 0.23 mg/g DW, respectively. These compounds might partly contribute to the antioxidant activity of *Akebia trifoliate* peel extract. Chlorogenic acid was one of the most abundant polyphenols in many natural products and exhibited antioxidant, anti-inflammatory as well as anticancer effects (such as anti-metastatic effects and anti-proliferative activity) [26,27,28,29]. Besides, clinical studies indicated that chlorogenic acid could decrease the risk of metabolic syndromes and chronic diseases (cardiovascular diseases, neurodegenerative diseases and liver diseases). Furthermore, chlorogenic acid also showed gastrointestinal protective and renoprotective effects [30]. Rutin might have potential for the treatment of ethanol-induced hepatotoxicity and exhibit a beneficial effect on regulating mitochondrial functions [31,32]. Moreover, rutin could be a neuroprotective agent, which improved the glutamate metabolism of brains in rats and prevented glutamate excitotoxicity [33]. In addition, ellagic acid could ameliorate features of metabolic syndrome [34]. Furthermore, ellagic acid could decrease the risk of diabetes mellitus by reducing oxidative stress, alleviating inflammation and improving insulin resistance [35]. These results hinted that the extract of *Akebia trifoliate* peel might also possess these health benefits and could be developed into a functional food for the prevention and management of some chronic diseases induced by oxidative stress. Of course, these functions of *Akebia trifoliate* peel extract need to be verified by animal and human experiments before the crude extract could be developed into functional food.

## 4. Conclusions

In this study, an MAE method was established for the extraction of natural antioxidants from *Akebia trifoliate* peel. Single-factor, as well as RSM experiments, were conducted to obtain optimal extraction conditions. The results of single-factor experiments showed that ethanol concentration, solvent-to-material ratio as well as extraction time were three main parameters affecting the extraction efficiency, and their interactions were further studied by RSM with CCD. The optimal conditions were as follows: 49.61% ethanol (*v*/*v*); solvent-to-material ratio, 32.57:1 mL/g; extraction time, 39.31 min; extraction temperature, 50 °C; as well as microwave power, 500 W. Under optimal extraction parameters, the TPC value of *Akebia trifoliata* peel extract was 33.17 ± 0.62 mg GAE/g DW, the FRAP value was 356.51 ± 3.06 µM Fe(II)/g DW and the TEAC value was 191.12 ± 3.53 µM Trolox/g DW. These actual values were close to the predicted values and certified the accuracy and reliability of the fitted model. Besides, compared with a conventional maceration extraction technique, the MAE effectively improved the extraction performance of natural antioxidants from *Akebia trifoliate* peel. Further, chlorogenic acid, rutin and ellagic acid in the extract were determined by the HPLC method, which might partly be responsible for the antioxidant activity of *Akebia trifoliate* peel. The extract could be used to develop functional food for the prevention and treatment of some diseases induced by oxidative stress. These results will be beneficial for the utilization of the by-product of *Akebia trifoliate* fruit. Since chlorogenic acid, rutin and ellagic acid showed many bioactivities, such as antioxidant, anti-inflammatory, anticancer and renoprotective effects, and is cardiovascular-protective, neurodegenerative-protective, hepato-protective and gastrointestinal-protective, and the crude extract of *Akebia trifoliate* peel contains these substances, the bioactivity and health benefits of *Akebia trifoliate* peel extract is worthy of being studied and certified in the future.

## Figures and Tables

**Table 1:** Selected variables as well as coded levels of CCD.

Selected Variables (Symbol)	Units	Coded Levels				
		−1.68	−1	0	1	1.68
Ethanol concentration (X_1_)	% (*v*/*v*)	33.18	40	50	60	66.82
Solvent-to-material (X_2_)	mL/g	13.18	20	30	40	46.82
Extraction time (X_3_)	min	23.18	30	40	50	56.82

**Table 2:** Response surface design and the actual response values of the extract.

Run	X_1_	X_2_	X_3_	Y (Actual Response Values)		
	Ethanol Concentration, % (*v*/*v*)	Solvent-to-Material mL/g	Extraction Time min	FRAP (µmol Fe(II)/g)	TEAC (µmol Trolox/g)	TPC (mg Gallic acid/g)
1	60 (1)	20 (−1)	30 (−1)	298.98	150.37	29.49
2	50 (0)	30 (0)	23.18 (−1.68)	337.76	172.84	31.95
3	50 (0)	30 (0)	56.82 (1.68)	316.47	163.84	31.25
4	50 (0)	30 (0)	40 (0)	343.04	178.13	30.58
5	50 (0)	13.18 (−1.68)	40 (0)	343.09	167.69	32.44
6	33.18 (−1.68)	30 (0)	40 (0)	324.19	162.56	31.85
7	50 (0)	30 (0)	40 (0)	347.47	197.13	32.18
8	60 (1)	20 (−1)	50 (1)	286.31	142.66	26.84
9	66.82 (1.68)	30 (0)	40 (0)	319.33	182.41	28.34
10	50 (0)	30 (0)	40 (0)	372.19	195.99	32.72
11	60 (1)	40 (1)	30 (−1)	274.34	115.56	27.17
12	50 (0)	46.82 (1.68)	40 (0)	324.02	176.56	30.69
13	40 (−1)	40 (1)	30 (−1)	317.01	167.98	30.08
14	50 (0)	30 (0)	40 (0)	376.76	193.56	33.04
15	50 (0)	30 (0)	40 (0)	369.61	197.70	32.06
16	40 (−1)	20 (−1)	50 (1)	325.46	152.66	30.42
17	40 (−1)	40 (1)	50 (1)	340.44	172.17	31.85
18	40 (−1)	20 (−1)	30 (−1)	355.65	169.23	32.04
19	60 (1)	40 (1)	50 (1)	325.01	187.22	32.55
20	50 (0)	30 (0)	40 (0)	369.04	198.56	31.03

**Table 3:** The results of ANOVA analysis for the quadratic models.

Response Values	Source	Sum of Squares	df	Mean Square	*F* Value	*p* Value	Significance
FRAP	Model	10,628.33	9	1180.93	7.28	0.0023	significant
	Residual	1622.44	10	162.24			
	Lack of Fit	1199.41	5	239.88	2.84	0.1387	not significant
	Pure Error	423.03	5	84.61			
	Cor Total	12,250.77	19				
	R^2^	0.8676					
	Adjusted R^2^	0.7484					
	C.V.%	3.78					
TEAC	Model	5420.07	9	602.23	6.52	0.0036	significant
	Residual	923.86	10	92.39			
	Lack of Fit	734.73	5	146.95	3.88	0.0813	not significant
	Pure Error	189.13	5	37.83			
	Cor Total	6343.92	19				
	R^2^	0.8544					
	Adjusted R^2^	0.7233					
	C.V.%	5.42					
TPC	Model	37.43	9	4.16	3.88	0.0230	significant
	Residual	10.72	10	1.07			
	Lack of Fit	7.29	5	1.46	2.13	0.2136	not significant
	Pure Error	3.43	5	0.6858			
	Cor Total	48.15	19				
	R^2^	0.7773					
	Adjusted R^2^	0.5769					
	C.V.%	3.32					

**Table 4:** The estimated coefficients and their statistical significance for quadratic model.

Model Parameter	FRAP		TEAC		TPC	
	Coefficient	*p* Value	Coefficient	*p* Value	Coefficient	*p* Value
X_1_	−8.28	0.0373	4.51	0.1136	−0.8292	0.0143
X_2_	0.0098	0.9775	10.06	0.0031	0.2212	0.4482
X_3_	−3.41	0.3454	−4.25	0.1336	−0.1026	0.7220
X_1_X_2_	10.10	0.0489	11.14	0.0083	0.8785	0.0373
X_1_X_3_	−0.2619	0.9548	−2.67	0.4508	−0.0665	0.8595
X_2_X_3_	8.76	0.0803	0.3095	0.9292	1.04	0.0177
X_1_^2^	−16.32	0.0007	−7.70	0.0125	−0.8696	0.0097
X_2_^2^	−12.78	0.034	−7.83	0.0114	−0.3293	0.2551
X_3_^2^	−15.56	0.0009	−9.16	0.0047	−0.3167	0.2726
Intercept	367.20		194.18		32.18	

**Table 5:** Comparison of the response values of fitted model prediction, actual experiment and maceration extraction.

Response Values	Microwave-Assisted Extraction		Maceration Extraction
	Predicted	Actual	
TPC (mg GAE/g DW)	32.23	33.17 ± 0.62	26.28 ± 0.38
FRAP (µM Fe(II)/g DW)	366.59	356.51 ± 3.06	297.66 ± 1.37
TEAC (µM Trolox/g DW)	196.18	191.12 ± 3.53	149.54 ± 1.87

## Data Availability

The data is kept in School of Public Health, Sun Yat-Sen University.
